# DVT方案治疗母细胞性浆细胞样树突细胞肿瘤1例报告并文献复习

**DOI:** 10.3760/cma.j.cn121090-20230524-00211

**Published:** 2024-01

**Authors:** 杰 施, 宁 徐, 艳 牛, 思寻 贾, 晨萌 杨, 美云 方

**Affiliations:** 大连大学附属中山医院血液科，大连 116000 Department of Hematology, Affiliated Zhongshan Hospital of Dalian University, Dalian 116000, China

## Abstract

母细胞性浆细胞样树突细胞肿瘤（BPDCN）是一种罕见的具有高度侵袭性的血液系统恶性肿瘤，目前无统一治疗方案，预后极差。大连大学附属中山医院报道1例85岁BPDCN男性患者应用DVT方案（地西他滨联合维奈克拉、沙利度胺）治疗获完全缓解的病例。患者皮肤结节起病，病理提示BPDCN，皮肤结节二代测序提示IDH2、ASXL1热点突变。地西他滨联合维奈克拉、沙利度胺的DVT非化疗治疗方案对于BPDCN疗效显著，起效快，缓解程度深，安全性好，尤其适用于无法耐受强化疗的老年患者。

## Abstract (en)

Blastic plasmacytoid dendritic cell neoplasm (BPDCN) is a rare and highly aggressive hematological malignancy, there is no standard treatment and the prognosis is very poor. Affiliated Zhongshan Hospital of Dalian University report a case of 85-year-old BPDCN male patient treated with DVT regimen (decitabine combined with Venetoclax and thalidomide) and achieved complete remission. The patient with skin nodules and the pathology diagnosed BPDCN, the next generation sequencing of skin nodules showed mutations of IDH2 and ASXL1. DVT (decitabine combined with Venetoclax and thalidomide) has significant efficacy with rapid and deep remission for BPDCN, and the adverse effects is less, especially suitable for elderly patients who cannot tolerate intense chemotherapy.

母细胞性浆细胞样树突细胞肿瘤（blastic plasmacytoid dendritic cell neoplasm，BPDCN）是一种罕见的具有高度侵袭性的血液系统恶性肿瘤，起源于浆细胞样树突细胞前体细胞，主要发生于皮肤，常累及骨髓，并呈白血病样播散。Adachi等[1]于1994年首先报道该病，2008年版世界卫生组织（WHO）造血与淋巴组织肿瘤分类[2]将其命名为BPDCN，并归类于急性髓系白血病和相关前体细胞肿瘤，2016年版WHO将BPDCN归为独立的一类疾病[3]。2022年版WHO造血与淋巴组织肿瘤分类将BPDCN归类于组织细胞/树突状细胞肿瘤中。本病目前无统一治疗方案，既往强化疗、造血干细胞移植治疗均疗效差，中位生存期不足2年[4]。我们报道1例高龄BPDCN应用DVT方案（地西他滨联合维奈克拉、沙利度胺）治疗获完全缓解的病例并作文献复习如下。

## 病例资料

患者，男，85岁，因“发现全身皮下结节5个月余”于2022年3月1日入院。患者2021年10月左颌下出现1处皮肤结节，大小约0.5 cm×0.5 cm，突出于皮肤表面，呈红色，质硬，边缘清楚，无疼痛及瘙痒，无发热、盗汗，无消瘦等不适。后皮肤结节逐渐增多，遍及颜面部、前胸后背、双下肢，均突出于皮肤表面，最大结节约4.0 cm×3.0 cm，最小结节约1.0 cm×1.0 cm。2021年12月辗转于当地多家医院就诊，予外用药膏局部治疗（不详），症状未见明显好转，全身皮肤结节逐渐增大，部分融合。2022年2月11日至22日于皮肤病专科医院住院，皮肤组织活检后予每日一次口服沙利度胺50 mg调节免疫，中药玉屏风辅助治疗，外用“复方曲安奈德、糠酸莫米松、复方维生素E乳膏”，局部涂擦，经治疗全身皮肤结节略有变小、变软。病理结果回报：表皮变薄，真皮及脂肪浅层间质、毛囊、血管周围可见致密的中等大小细胞浸润，胞质嗜碱性，形态单一，核仁较小，可见核分裂象；免疫组化结果显示：CD4、CD56、CD123、TCL-1、E2-2为阳性，CD3散在阳性，MPO、CD20、CD63、CD163、TDT阴性。病理诊断：BPDCN。为进一步诊治，患者2022年3月1日首次于我科住院。既往史：高血压病50余年，血压最高达200/100 mmHg（1 mmHg＝0.133 kpa），每日一次苯磺酸氨氯地平5 mg降压治疗，监测血压130/80 mmHg。无血液病家族史。查体：体温正常，血压130/65 mmHg，体型消瘦，皮肤结节遍及颜面部、前胸后背、双下肢，结节均突出于皮肤表面，呈圆形，暗红色、褐色，质硬，边缘清楚，其中最大者4.0 cm×3.0 cm，最小者1.0 cm×1.0 cm，胸锁乳突肌双侧后缘可触及多枚肿大淋巴结，最大者1.0 cm×1.0 cm，双侧腋窝可触及多枚肿大淋巴结，左侧最大者3.0 cm×2.0 cm，右侧最大者1.5 cm×1.5 cm，双侧腹股沟可触及多枚肿大淋巴结，左侧大者1.0 cm×1.0 cm，右侧大者2.0 cm×1.0 cm，以上淋巴结均质韧，边界清晰，可活动，肝脾肋缘下未触及。辅助检查血常规：WBC 2.09×10^9^/L，中性粒细胞计数0.85×10^9^/L，HGB 133 g/L，PLT 119×10^9^/L。头部CT平扫未见占位，胸部CT及全腹平扫提示纵隔、肺门、腹膜后无肿大淋巴结。骨髓细胞形态学：骨髓增生活跃，粒系占21.50％、红系占31.50％、粒系/红系比为0.68/1，原始细胞占28.00％，形态：胞体中等大小、不规则，核圆形或不规则形、核仁隐约可见，胞质灰蓝色、量丰富、无颗粒，易见伪足突出（图1）。MPO染色：病理细胞100％阴性。骨髓流式细胞术检测免疫表型：异常细胞群约占有核细胞的10.17％，表达CD123、CD303、CD304、CD56、CD2、CD45RA；弱表达CD4、CD38、CD11c；不表达TCL-1、CD117、CD1c、CD34、CD141、TDT、MPO、CD19、CD8、CD10、CD5、CD23、FMC7、CD79b、CD3、CD200、CD103、CD25、CD22、IgD、IgM、CD20、Kappa、Lambda、TCR g/d、CD7、CD57、CD45RO、GrenzymeB、Perforin、CD161、CD94。左侧腋窝淋巴结病理及免疫组化结果符合BPDCN表现。外院皮肤结节病理见图2，补充免疫组化：BCL2^+^（>80％），CD38^−^，CD303个别^+^，原位杂交：EBER^−^。皮肤结节FISH：TP53、MYC基因未见异常。皮肤结节二代测序：与疾病密切相关的热点突变位点检测结果：IDH2的Exon4 c.419G>A p.R140Q突变频率为38.6％，ASXL1的Exon12 c.1902_1924del23 p.E635Rfs*15突变频率为21.9％。诊断：BPDCN（累及皮肤、淋巴结、骨髓）。我们根据二代测序结果结合该患者超高龄，选用地西他滨+维奈克拉+沙利度胺的DVT方案治疗，于2022年3月9日开始治疗，具体方案如下：第1～5天，地西他滨10 mg/d，维奈克拉100 mg/d；第6～14天，维奈克拉200 mg/d；第1～21天，沙利度胺50 mg/d，28 d为1个疗程。治疗第3天，皮肤结节逐渐消退，淋巴结逐渐缩小（图3），2个疗程后血常规正常，皮肤结节全部消退，遗留色素沉着，肿大淋巴结消退，骨髓肿瘤细胞0.2％，获得完全缓解，且治疗耐受性好，骨髓抑制轻，未发生感染及出血不良反应。现已完成5个疗程治疗，持续完全缓解，继续治疗随访中。

**图1:**
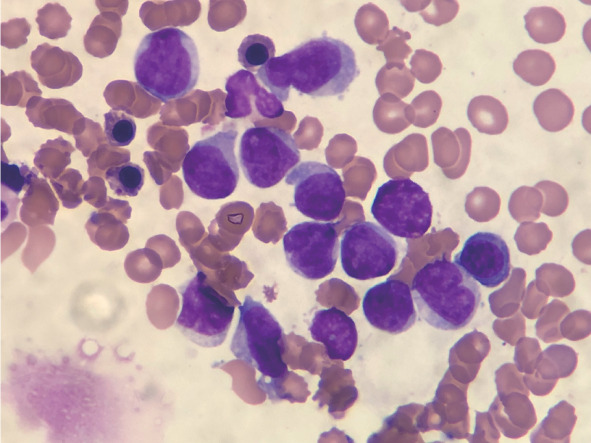
骨髓形态学（瑞氏-吉姆萨染色，×100） 注 骨髓可见异常细胞，胞体中等大小、不规则，核圆形或不规则形、核仁隐约可见，胞质灰蓝色、量丰富、无颗粒，易见伪足突出

**图2:**
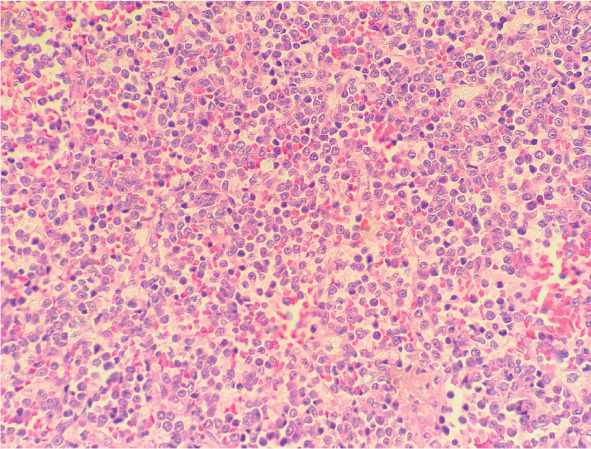
皮肤病理（HE染色，×400） 注 真皮内可见异常细胞片状增生，胞体大，胞质少，染色质细致

**图3:**
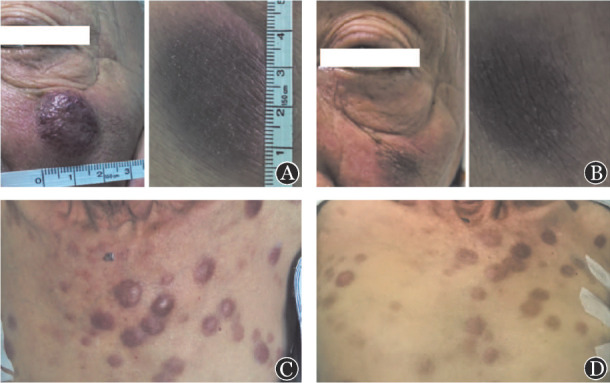
治疗前后皮肤结节临床表现 **A** 治疗前颜面部皮肤结节；**B** 治疗第3天颜面部皮肤结节；**C** 治疗前皮肤结节；**D** 治疗第3天皮肤结节

## 讨论及文献复习

BPDCN是一种罕见的具有高度侵袭性的血液系统恶性肿瘤，起源于浆细胞样树突细胞前体细胞，主要发生于皮肤，常累及骨髓，并呈白血病样播散。BPDCN发病率不明，有报道显示每年大约为0.04/10万[5]，老年人多见，诊断时中位年龄60～70岁，男女比例为3∶1[4]。目前发病机制尚未明确。

BPDCN常见的临床表现是皮肤病变，大约90％的患者皮肤受累，多表现为孤立或多发性红斑、结节，也有部分患者仅表现为皮疹，往往就诊于皮肤科[6]。骨髓、淋巴结和脾脏常受累。大约30％的患者累及中枢神经系统[6]–[7]。

BPDCN的肿瘤细胞为幼稚细胞，核不规则，染色质细，核分裂易见，形态无特异性，容易被误诊为其他更常见的血液系统恶性肿瘤，明确诊断通常取决于免疫表型，BPDCN的肿瘤细胞表达CD4、CD56、CD123、CD303，其中CD123是白细胞介素3受体亚基α，几乎所有BPDCN都过表达CD123，其他谱系特异性标志，如MPO、CD13、CD64、CD19、CD20、CD79a、CD3均阴性，且不表达CD34，如果5个主要细胞表面抗原（CD4、CD56、CD123、TCL-1、CD303）中的4个表达阳性，则可以确诊BPDCN[8]。细胞遗传学：BPDCN无特征性的细胞遗传学标志，三分之二的BPDCN患者存在异常核型，最常见的细胞遗传学异常包括5q、6q、12p、13q和15q异常及9号染色体单体[9]。分子生物学异常：可见MYC基因重排[10]，发生率为10％～15％，与预后差相关。二代测序常见TET2、ASXL1、TP53、NPM1突变[11]–[12]。2019年，Sapienza等[13]使用全外显子组测序发现参与表观遗传调控的基因是最常见的基因异常，包括DNA甲基化和组蛋白修饰相关基因。

BPDCN为罕见病，目前对该病的认识多来自病例报告，尚缺乏标准的治疗方案，目前常采用急性髓系白血病（AML）或急性淋巴细胞白血病（ALL）样的治疗方案，文献报道ALL样的治疗方案缓解率高于AML方案[14]–[15]，但治疗反应往往是短暂的。Laribi等[16]回顾性分析了来自75个中心的398例BPDCN患者的临床资料，这是迄今为止最大的一项针对BPDCN的回顾性研究，其中129例患者接受非霍奇金淋巴瘤样方案，113例患者接受急性白血病样方案，61例和16例患者化疗后分别进行了异基因和自体造血干细胞移植（HSCT），27例接受放射治疗，6例接受新药物治疗，62例接受姑息治疗。中位随访12（0.2～137）个月，中位总生存（OS）期为18（95％ *CI* 15～22）个月。接受非霍奇金淋巴瘤样或急性白血病样治疗方案后进行异基因HSCT的患者，预后最好，未达到中位OS期，提示HSCT有可能改善BPDCN的预后，但BPDCN多见于老年人，限制了移植的临床应用。参与DNA甲基化途径的基因突变通常与BPDCN预后不良相关。Sapienza等[13]对14例BPDCN患者进行全外显子组测序，发现表观遗传基因是BPDCN的潜在治疗靶点，他们在小鼠模型中证明了去甲基化药物阿扎胞苷和地西他滨可控制疾病进展。另有研究表明，阿扎胞苷单药治疗BPDCN有效，但不能达到长期完全缓解[17]–[18]。BCL2在正常的浆细胞样树突细胞中不表达，但在大多数BPDCN中过表达[19]–[20]。Montero等[21]通过体外和体内实验证实了BPDCN细胞的生存依赖于BCL2蛋白，对BCL2抑制剂维奈克拉敏感，随后在2例复发难治的BPDCN患者中证实了维奈克拉的显著疗效。Agha等[22]报道了1例BPDCN应用维奈克拉治疗成功的患者，其皮肤结节免疫组化BCL2过表达，予维奈克拉400 mg每日口服，治疗4周后骨髓活检未发现肿瘤细胞，治疗6个月后PET-CT提示完全缓解，因此BCL2抑制剂可能成为治疗BPDCN非常有前景的药物之一。Le Calloch等[23]首次应用维奈克拉联合阿扎胞苷一线治疗1例87岁高龄BPDCN患者，3个疗程后达完全缓解，OS期达1年，提示去甲基化药物联合BCL2抑制剂方案可能优于单药治疗。Mirgh等[24]报道1例75岁诊断BPDCN后应用阿扎胞苷联合维奈克拉方案复发的患者，更换达雷妥尤单抗联合硼替佐米方案，4个疗程后完全缓解，且耐受性良好，提示达雷妥尤单抗联合硼替佐米治疗BPDCN可能有一定疗效。由于BPDCN肿瘤细胞均过表达CD123，因此CD123是重要的治疗靶点，Frankel等[25]开展了针对BPDCN的首个前瞻性研究，11例患者应用SL-401（靶向CD123的融合蛋白）治疗，在9例可评估疗效的患者中，1个疗程治疗后，5例患者达到了完全缓解，2例达到了部分缓解疗效。在另一项Tagraxofusp治疗47例初诊或复发难治BPDCN患者的临床研究中，其中29例为初诊患者，72％（21例）达到了完全缓解疗效，45％（13例）患者缓解后进行了HSCT巩固治疗（10例异基因和3例自体），18个月OS率为59％，24个月OS率为52％。在15例复发/难治BPDCN患者中，总体缓解率为67％，中位OS期为8.5个月[26]。在初治和复发患者中，Tagraxofusp均显示出了明显的疗效，因此2018年12月，美国食品药品监督管理局（FDA）批准了Tagraxofusp（SL-401）用于治疗BPDCN，这是第一个针对BPDCN的靶向药物[27]。

本病例的特殊性在于患者为85岁的高龄老年人，以皮肤结节为首发表现，伴有淋巴结和骨髓受累。BPDCN肿瘤细胞通常对常规化疗耐药，既往强化疗、HSCT治疗均疗效差，靶向药物如CD123单抗和BCL2抑制剂、去甲基化药物显示出疗效，而针对CD123的靶向药物目前在我国尚未上市，患者二代测序发现表观遗传学调控基因突变，超高龄患者无法耐受急性白血病样的强化疗方案，选用地西他滨联合维奈克拉、沙利度胺的DVT非化疗治疗方案，疗效显著，不仅起效快，且缓解程度深，治疗期间未发生严重骨髓抑制及感染等并发症，尤其适用于无法耐受强化疗的老年患者，该患者继续治疗及随访中。
